# Editorial

**Published:** 1849-10

**Authors:** 


### THE MEDICAL EXAMINER.

PHILADELPHIA, OCTOBER, 1849.

### EPIDEMIC CHOLERA IN PHILADELPHIA.

The Board of Health of this city having presented their official report of the number of cases of cholera, and the deaths therefrom, during its recent visitation, we are now able to lay before our readers a comparison of this epidemic with that of 1832.

We would, at the same time, respectfully suggest to the editors of the various medical journals throughout the country, that a table of similar character, prepared by them, would constitute as a whole, an exceedingly valuable statistical account of the disease as it has recently visited us.

It must be premised, that there are certain sources of error that unavoidably find their way into the reports of our official bodies notwithstanding all the care that may be observed in their preparation. Among these it may be mentioned that many cases are not reported at all; and on the other hand, that during the prevalence of such an epidemic, charlatans and irregular practitioners report everything as cholera, in hopes of extending their own reputation by spreading their marvellous histories of cures. Among the interments in our city, are likewise included those of bodies brought from the country. With these facts before us, then, it will be understood that the following account is merely the nearest approximation to the truth that can be obtained under the circumstances.

The first official bulletin was issued on May 30th, and the last on August 19th, the board of health having declared at the latter date that cholera was no longer epidemic. The whole period of its duration was 82 days, during which time there were reported 2131 cases, and 744 deaths, or one death in 2.86 cases. On the 14th July the greatest number of deaths occurred, viz., 32; cases 84; and on the 29th of July the greatest number of cases, viz., 90; deaths 24. The increase on the last date was owing to the violent breaking out of the disease in the Alms House.

During the months of May and June there were reported 278 cases and 97 deaths, or one death in 2.86 cases. In July 1566 cases, and 578 deaths, or 1 in 2.70. In August 277 cases, and 69 deaths, or 1 in 4.01 cases.

The whole population of Philadelphia, city and county included, amounts to about 350,000. This will give a ratio of cases to population of 1 to 164.24. The ratio of deaths to cases during the whole epidemic was 1 to 2.86 cases, and the ratio of deaths to population was 1 to 470.4 inhabitants.

| Locality. | Population. | Cases. | Deaths. | Ratio of cases to population | Ratio of deaths to cases. | Ratio of deaths to population. |
|---|---|---|---|---|---|---|
| | | | 1849. | | | |
| Philada., | 350,000. | 2131. | 744. | 1 in 164.24 | 1 in 2.86 | 1 in 470.4 |
| | | | 1832. | | | |
| " | 160,000. | 2314. | 754. | 1 in 69.1 | 1 in 3.06 | 1 in 212.2 |

By a comparison of the above tables it will be seen, that while the whole number of cases and deaths was more than half less in 1849 than in 1832, the mortality was much the same in the two epidemics.

As in 1832, the epidemic was not confined to any one part of the city, but was extended to every portion. Yet strange to say, in many localities, where from the filth and depraved condition of their inhabitants almost entire depopulation was anticipated, it scarcely appeared at all.

The total number of cases occurring in private practice amounted to 1439, the deaths to 386, or 1 to 3.7. In Hospital practice 692 cases, 357 deaths, or 1 death to 1.9 cases.

The following table shows the number of cases and deaths in the city proper, the districts, and hospitals.

*Total of Cases during the prevalence of the Epidemic.*

| Districts. | Cases. | Deaths. |
|---|---|---|
| City, - - - - - | 388 | 127 |
| Southwark, - - - - | 296 | 50 |
| Kensington, - - - - | 218 | 54 |
| Spring Garden, - - - | 108 | 33 |
| Moyamensing, - - - | 191 | 52 |
| Northern Liberties, - - | 147 | 38 |
| Penn District, - - - | 14 | 4 |
| Richmond, - - - - - | 39 | 13 |
| West Philadelphia, - - | 22 | 11 |
| Passyunk, - - - - - | 10 | 3 |
| Residence not stated, - - | 6 | 1 |
| Hospitals. | | |
| Cherry street, - - - | 35 | 19 |
| Pine street, - - - - | 9 | 4 |
| South street, - - - - | 3 | 1 |
| Southwark, - - - - | 60 | 9 |
| Kensington, - - - - | 14 | 6 |
| Spring Garden, - - - | — | — |
| Moyamensing, - - - | 106 | 26 |
| Northern Liberties, - - - | 47 | 22 |
| Penn District, - - - | — | — |
| Richmond, - - - - | 13 | 3 |
| West Philadelphia, - - - | 2 | 1 |
| Bush Hill, - - - - | 26 | 16 |
| Alms House, - - - - | 314 | 229 |
| County Prison, - - - | 63 | 20 |
| Coroner, - - - - | 1 | 1 |
| Total, | 2131 | 744 |

The above statistics were obtained from the bulletins issued daily from the Health Office. The sources of error above alluded to, of course prevail here, making it exceedingly difficult to arrive at the facts of the case. In the following table, the total number of *interments** from cholera up to September 8th, is exhibited, amounting to 1012. During the epidemic, (that is, from May 30th, to August 19th,) the number of deaths from cholera, according to this report, amounted to 962. The discrepancy in this case is owing to the facts that daily reports were

*For this table we are indebted to Mr. Samuel Marks, Clerk of the Board of Health.

not made by many physicians, that several cases were reported by the coroner, and that some of the interments were of persons brought from the country and interred within the city limits, while no reports were made of their deaths to the Board of Health. It will be seen also, that deaths from cholera continued to be reported until September 8th, although the Board no longer issued bulletins, they not considering the disease to be epidemic, although it was endemic in certain localities, there having been in one block 15 deaths in the week, ending September 1st.

The following table drawn from Dr. Jackson's report in February, 1833,* exhibits the number of deaths at corresponding ages, and the ratio of males to females.

| AGES. | | NUMBER OF DEATHS. |
|---|---|---|
| Under 1 year, | | 4 |
| Between 1 and 2 years, | | 4 |
| 2 | 5 | 30 |
| 5 | 10 | 39 |
| 10 | 15 | 19 |
| 15 | 20 | 22 |
| 20 | 30 | 179 |
| 30 | 40 | 228 |
| 40 | 50 | 159 |
| 50 | 60 | 100 |
| 60 | 70 | 71 |
| 70 | 80 | 47 |
| 80 | 90 | 5 |
| 90 | 100 | 1 |
| 100 | 110 | 1 |

In relation to sexes the deaths presented the following ratio:—

| | MALES. | FEMALES. |
|---|---|---|
| | 539 | 370 |
| Under twenty years | 70 | 48 |

This report of the Board of Health includes only the well marked cases of cholera; but before, and during the continuance of the disease, affections of the stomach and bowels were unusually prevalent, a general irritability of the whole alimentary canal existed, which rendered it much more liable to disturbance from slight causes than in ordinary times. These mild attacks, usually yielded readily to prompt and early treatment, and in this way the number of cases and deaths was kept down to the present low figure. This condition of the animal economy throughout the whole community, was doubtless to be attributed to the operation of the epidemic influence upon them, and it affords abundant evidence of its wide spread existence among us.

We do not propose to enter into any detail of the symptoms, progress, pathology, or treatment of the disease, having already done tha in a previous number, (July,) and the latter department (treatment) having been ably discussed from time to time in the preceding pages. Our object is simply to present the *statistics* of the disease in Philadelphia to our readers, having done which our task is ended.

* Vide Amer. Jour. of Med. Sciences, Feb. 1833. Personal observations and experience of epidemic or malignant cholera in the city of Philadelphia, by Samuel Jackson, M. D., Assistant to the Professor of Institutes and Practice of Medicine and Clinical practice in the University of Penn'a.

OBITUARY.

It is with deep regret we learn the death of *Dr. J. P. Harrison*, of Ohio, one of the Editors of the Western Lancet, of *Dr. A. Brigham*, Superintendent of the N. Y. State Lunatic Asylum, and *Dr. John Butterfield*, Professor of Theory and Practice of Medicine in Starling Medical College, and Editor of the Ohio Medical and Surgical Journal. All were prominent and greatly respected members of our profession. The former will be remembered as having been one of the Vice Presidents of the American Medical Association at its last meeting, and as chairman of the Committee on Medical Literature. He died on the 1st of September, of cholera, after an illness of only a few hours. He was a man of most unbounded zeal, an industrious and faithful student, an honorable and high minded gentleman, and withal a Christian. In his editorial capacity he was ever courteous, dignified and impartial.

The name of Dr. Brigham has long been connected with the management and treatment of the insane. He was for a long time physician to Hartford Retreat, from which he was appointed to take charge of the N. Y. State Asylum at Utica, the organization of which he conducted. He was extensively known as the author of several admirable works, viz: *The Influence of Religion on Health; The influence of Mental Cultivation and Mental Excitement;* a work on *Diseases of the Brain;* and as Editor of the Journal of Insanity. The proceedings of the conventions of Superintendents of Insane Asylums teem with the fruits of his great experience, always characterized by strong good sense and philanthropy. He was a zealous advocate of Phrenology, which he greatly cultivated.

Dr. Butterfield had been failing in health for some time, but still continued his editorial duties, in the conduct of which he manifested singular tact and judgment. Under his fostering care the journal had steadily increased in favor and extension, and bid fair, though but of recent origin, to be one of the most acceptable and useful periodicals of the day.

From England we hear of the death of *Sir Charles Scudamore*, and *Dr. John Reid.** The latter was well known as the industrious author of the "Physiological, Anatomical, and Pathological Researches," as well as of numerous scientific papers which appeared at different times in the Journals. His published works speak for the manly vigor of his mind. He died at the age of 40 of cancer of the tongue. Those who

* London Medical Times.

have watched the progress of modern physiology will know that one of the brightest minds of the present generation has passed away.

---

SOUTHERN MEDICAL REPORTS.

From a circular lately received we learn with pleasure that *Dr. E. D. Fenner* proposes to publish an annual volume on the meteorology, medical topography, and diseases of the Southern States, an undertaking for which he is eminently qualified.

The object of this work, is to collect and present, in a *durable form*, the observations of physicians residing in different parts of the Southern States, with the view to the cultivation of medical science, and the formation of the medical history of the times.

It will consist of GENERAL and SPECIAL REPORTS ; the first to contain concise accounts of the *meteorology, medical topography and prevailing diseases* throughout the year ; the second will be devoted to *extraordinary cases, surgical operations, &c.*

These reports are all to be handed to the Editor, by the first of January, and will appear in a *neatly bound volume*, as soon thereafter as the work can be done. Each volume will also contain a brief retrospect of the latest discoveries and improvements contained in the Medical Journals of the year.

The cost of this work will be in proportion to the extent of patronage. It is contemplated that each volume will contain from *five to six hundred pages*, and will be furnished to subscribers at $3.50. One copy will be sent gratuitously to all contributors, and three or more copies to the authors of annual reports.

All Medical Societies within the above limits, are respectfully invited to send in condensed reports of their transactions during each year.

Publishers are requested to forward copies of all new medical books; in return for which, bibliographical notices will be given.

Medical Colleges throughout the Union, are requested to forward their annual circulars ; from which will be extracted statistics, to show the progress of medical education. In short, the proposed work is designed to make up the medical history of the time, and to promote the cultivation of medical science in the Southern States. The Editor will do all in his power to render the work advantageous, as well to the collaborators as to the profession at large.

E. D. FENNER, M. D.,

*New Orleans, La., June* 1, 1849. *No.* 5, *Carondelet Street.*

☞ Due notice will be given when the work is completed, and it will be deposited at the principal commercial points in the Union.

---

MODE OF PRESERVING GUM ELASTIC CATHETERS IN WARM CLIMATES.

Two suggestions as to the means of preserving catheters, have been sent to us since the publication of the article on that subject in the last number. One is to "coat them over with collodion," the other, to suspend them to a joist, enveloped as loosely as possible in paper, to prevent injury by flies, dust, &c. The latter plan has been found to succeed.

---

INCREASE OF THE CHOLERA IN EUROPE.

From the daily prints we glean the following items of intelligence. The cholera was greatly increasing in England. The deaths for the week ending 8th ult., in London, were 7796, of which 1663 were of cholera. In Liverpool, the deaths by cholera were said to be greater, in proportion, than in any part of England.

In Dublin it was on the increase.

Several distinguished persons have died of cholera in Paris and in other parts of France.

Vienna and Berlin are at the present time suffering more than Paris. At Berlin the deaths are more than forty per day.

---

BOARD OF EXAMINERS—MEDICAL DEPARTMENT, U. S. A.

The following gentlemen will constitute the Board of Examiners about to meet in this city, October 15th: Surgeons Mower and Satterly, and Assistant Surgeon Southgate. There is another vacancy by a recent death.

